# Predictors of long-lasting insecticide-treated bed net ownership and utilization: evidence from community-based cross-sectional comparative study, Southwest Ethiopia

**DOI:** 10.1186/1475-2875-12-406

**Published:** 2013-11-09

**Authors:** Lelisa D Sena, Wakgari A Deressa, Ahmed A Ali

**Affiliations:** 1 Department of Epidemiology, College of Public Health and Medical Sciences, Jimma University, Jimma, Ethiopia; 2 Department of Preventive Medicine, School of Public Health, College of Health Sciences, Addis Ababa University, Addis Ababa, Ethiopia

**Keywords:** Gilgel-gibe, Long-lasting insecticide-treated bed nets/insecticide-treated bed nets, Malaria, Ownership, Possession, Utilization

## Abstract

**Background:**

Malaria is the notorious impediment of public health and economic development. Long-lasting insecticide-treated bed nets/insecticide-treated bed nets (LLINs/ITNs) are among major intervention strategies to avert the impact the disease. However, effectiveness of LLINs/ITNs depends on, *inter alia*, possessing sufficient number, proper utilization and timely replacement of nets. Thus, the World Health Organization (WHO) recommends surveys to evaluate possession and proper use of LLINs/ITNs by households.

**Methods:**

A cross-sectional comparative household survey was conducted during peak malaria transmission season using interviewer-introduced questionnaires in southwest Ethiopia. A study site was selected from villages around a man-made lake, Gilgel-Gibe (GG) and a control site, with similar geographic and socio-economic features but far away from the lake, was identified. A total of 2,373 households from randomly selected cluster of households were included into the study and heads/spouses of the households responded to interviews. Binary and multinomial logistic regressions were used to identify predictors of LLIN ownership and utilization.

**Results:**

LLIN/ITN ownership among the study populations was 56.6%, while 43.4% of households did not own a net. A higher proportion of households in GG reported owning at least one LLITN/ITN compared to control village (OR =2. 2, P <0.001) and more households in GG reported having only one LLITN/ITN in contrast to households in the control village (OR = 2.1, P <0.001). The mean number of LLINs/ITNs owned was 1.6 for GG residents and 1.8 for control village with a mean difference of -0.26 (95% CI = - 0.34, -0.19). The age of household heads, household relative wealth index (RWI), distance to nearest health service and accessibility to transportation showed a significant association with ownership of LLINs/ITNs. The probability of owning two or more LLINs/ITNs was positively associated with age of household head. Marital status of household heads, RWI, distance to nearest health service, accessibility to transport, residence and household size showed a significant association with utilization of LLINs/ITNs.

**Conclusion:**

Attention needs to be given to the poor, distant and inaccessible households in the efforts of malaria intervention programmes, such as free distribution of LLINs/ITNs. Well-tailored information, education and communication is needed to address the problem of non-users.

## Background

Until recently, malaria has been a major public health problem in Ethiopia, causing millions of cases and thousands of deaths annually [1,2]. Present malaria control strategies involve early diagnosing and treating infected individuals and reducing human-mosquito contact rates through vector control efforts [3]. Long-lasting insecticide-treated bed nets/insecticide-treated bed nets (LLITNs/ITNs) are considered to be one of the major components of the selective vector control strategies in Ethiopia [4,5]. Since, 2005 the government of Federal Democratic Republic of Ethiopia embarked on a scaling-up of LLINs/ITNs where the coverage was planned to be 100% by the end of 2007 inline to the Roll Back Malaria recommendation of universal coverage [6,7].

However, bed nets as a tool for malaria control can present challenges, such as coverage, proper use and replacement of old and torn nets [8]. The possible shift in local malaria epidemiology prompts also the need for evaluation of their proper use and effectiveness in ensuring the long-term benefit of this control method [9-11]. According to the Ethiopian malaria indicator survey of 2007, the coverage in sampled malarious areas was 65.6% for households with one bed net and among those LLINs/ITNs-owning households only 53.2% of family members had slept under a LLIN/ITN the previous night of the survey [12]; this shows that both coverage and utilization were below the set targets [13,14]. Also, studies conducted in eastern and western part of the country showed that the coverage and utilization of existing LLINs/ITNs even by the vulnerable members (pregnant women and children) of the households were still very low [15,16].

According to local health offices information, the last distribution of LLINs to the present study areas was carried out in 2009 on free basis from district health offices to each household. However, assessment on possession and utilization of the LLINs was not done since then in this area. The World Health Organization (WHO) [17] recommends periodic household surveys to assess whether populations at risk receive sufficient LLINs/ITNs, that there is proper use of LLINs/ITNs. The present study was conducted to assess net possession, proper utilization and sufficiency of existing nets for all family members.

## Methods

### Study settings

This cross-sectional comparative study was conducted in two rural settings; one site was near to a man-made lake, Gilgel Gibe Hydroelectric Dam (GGHD), and a control site from an area distant from the lake. The site near GGHD is expected to increase the risk of malaria infection due to the man-made lake and hence this exposure in turn increases awareness of the residents about malaria risk and its control interventions [18]. The distant site from the artificial lake has not been undergone environmental modification like GGHD and would not be expected to affect the awareness level of the resident regarding malaria control intervention; hence, it is considered to be a control village.

The main socio-economic activities of both communities (villages) are mixed farming, including the cultivation of staple crops, and raising of cattle and small stock. All the villages are accessible to primary health care services (a health post for each *kebele*/village and one health centre in common). The villages were selected because of their similarity of geographic features within the Dam area with regard to altitude and presence of rivers, and equivalent range of distance from water bodies as a main risk of malaria.

GGHD is located 260 km southwest of Addis Ababa, the capital of Ethiopia, at 07.4253° to 07.5558° N and 037.1153° to 037.2033° E. The area lies between 1,734 and 1,864 m above sea level, which is within the range of mosquito-breeding altitude. Surveys conducted in the area [9,19,20] have shown that the prevalence of malaria in children was higher by many folds than the national malaria indicator survey results of 2007 [14]. The study households were selected from those that live within a 10-km radius of this artificial lake.

The control villages were identified from Kersa District, which is located adjacent to the GGHD on the west side. All the control villages are more than 15 km away from the lake so that the possible effect of the dam would not be reflected in the control area. This study considered household as a unit of study. The respondents to the survey were heads/spouses of households from the sample clusters (*gots*) consisting of about 30 households each.

### Sample size determination and sampling technique

The number of households used for study was calculated using two population proportion formulae [21,22] taking into account the two study sites (GGHD and control villages). From previous study [9], the proportion of households with at least one LLIN/ITN in GGHD village was 77%; assumining 95% confidence interval and power of 80% to detect a difference of 4% between proportions of LLINs/ITNs owing households in the two sites the sample size was calculated. Considering a 10% contingency for non-respondents, the number of households included in the survey was estimated to be 1,186 households from each site which implied a total of 2,372 households.

Initially, both villages (GGHD and control villages) were arranged into clusters (*gots*) based on their distances from the GGHD and other water bodies (streams, rivers, ponds, marshy lands) as possible mosquito-breeding sites. The total number of clusters was selected based on the assumption that each cluster (*got*) consists of 25–30 households according to current administrative structure). The equivalent number of clusters was categorized as near to and distant from water bodies based on maximum mosquito flight range [23]. The study clusters were selected randomly both from those near and distant *gots*.

### Conducting the survey

Household surveys were conducted during malaria peak transmission season in November 2012, on selected households. Semi-structured questionnaires are usually considered best for determining the frequency of beliefs, opinions, views, perceptions, and reported practices pertaining to malaria prevention and control, upon which more valid generalizations are possible [24-27]. Data were collected regarding socio-economic and LLIN possession and utilization. A questionnaire was developed by reviewing relevant literature, such as malaria indicator survey documents [28-31].

### Data management and analysis

Data were checked by investigators for correct completion of questionnaires. The edited data were entered into EPI data and exported to SPSS Statistics software version 20 for Windows. Data cleaning, recoding and restructuring of some variables to make them amenable for analysis (data preparation) was carried out using SPSS Statistics software. Durable household assets, sanitation facilities, farm animals, grain produced in the preceding year of the survey, housing conditions and vehicles were considered in the construction of household relative wealth index (RWI) using principal component analysis.

Socio-economic and demographic characteristics of study subjects were summarized in a Table and compared on the basis of means of continuous variables such as age, family size and income, using *t*-test for differences of means. Multinomial logistic regression was used to analyse factors associated with possession of LLINs and the number of LLINs owned. Factors affecting utilization of LLINs were analysed using binary logistic regression. To ensure the data quality, the data collection questionnaire was pretested; checklists and field guidelines were used. Adequate training was given to data collectors and field supervisors and job supervision was carried out daily by field supervisors.

### Ethical considerations

Ethical clearances were obtained from the Addis Ababa University College of Health Sciences Ethical Review Board and Oromia Regional Health Bureau. Cooperation and community consent was requested by formal letter and obtained from Jimma Zonal Office, District Health offices and local administrators; verbal consent was obtained from study subjects. Individuals who complained of fever were treated based on Ethiopian Malaria Diagnosis and Treatment Guideline [1]. Individual information was kept confidential: the names of individuals were removed from completed questionnaires and identified only by number; hard copies of the information were kept in a locked cupboard and the results were summarized in an aggregated manner.

## Results

A total of 2,373 (100%) individuals responded to the survey; 1,187 (50%) were from GG and 1,186 (50%) were from the control villages, corresponding to the number of households 5,946 (49.8%) and 5,984 (50.2%) respectively; and had similar sex compositions. The majority of heads of household in both sites were married although the figure was higher in control village (88.8 *vs* 86.5%); close to 9% of household heads from both study areas reported that they were widowed and about 1% of them were divorced. About three-quarters of heads of households, 77.4% GG and 73.4% control, were illiterate. Those that attended elementary (grades 1–8) constituted 11.5% from GG and 17.2% from control areas. Below 3% of heads of households attended grade 9 or more in both study sites. With regard to occupational status, most were farmers: 89.2% from control villages exceeded that of GG (65.3%) by about a quarter. Accessibility to health services in terms of distance, all of the control population reported to be within an estimated distance of 2 km of health services, in contrast to the GG population of which 19.2% reported living more than 2 km from health services. There was a similarity with regard to accessibility to vehicle transportation: about two-thirds (60.4% of GG and 62.2% of control) of the study populations were inaccessible to all-weather roads to reach health services.

The average age of household members was about 21 years, for both populations. Significant differences were not seen between the two populations regarding household size, average age of the populations, perceived monthly expenditure, number of farm animals owned, and amount of grain produced in one year preceding the survey. However, differences were observed pertaining to age of household heads where that of GG was higher by about two years, and perceived monthly income and relative wealth index. In both cases the control population seemed better off (Table 1).

**Table 1:** Comparison of study populations on the basis of demographic and economic characteristics of household, Southwest Ethiopia, November 2012

**Parameter**	**GG area mean (SE)**	**Control area Mean (SE)**	**Mean difference (SE)**	**t-statistic**	**95% CI**
Household size	4.95 (0.06)	5.06 (0.06)	-.104 (0.09)	- 1.2	-.274, .067
Household heads’ age	44.4 (0.4)	42.4 (0.4)	2.1 (0.59)	3.5	0.91, 3.2*
Family members’ age	20.96 (0.24)	20.63 (0.22)	0.33 (0.32)	1.03	-0.20, 0.96
Monthly income	309.5 (6.5)	431.9 (8.8)	-122.3 (10.9)	- 11.2	-143.8, -100.9*
Monthly expenditure	263.6 (5.5)	357.8(7.6)	-94.2 (9.4)	- 10.0	-112.6, 75.7
Farm animals owned	6.6 (0.1)	7.06 (0.13)	-0.47 (0.2)	- 2.6	-0.83, 0.11
Grain produced (quintal)	8.5 (0.2)	8.8 (0.16)	-0.4 (0.2)	- 1.7	- 0.81, 0.06
HH relative wealth index	- 0.13 (0.8)	0.12 (0.8)	- 0.6 (0.1)	- 2.3	- 0. 47, - 0.04*

GG = Gilgel-Gibe; SE = standard error of the mean difference; quintal = 100 kg;

*Significant at 0.05.

The overall LLIN ownership among the study populations was 56.6% (66.3% of GG, 46.8% of control) whereas 43.4% (33.7% of GG, 53.2% of control) reported that they did not own a net. A higher proportion of GG households reported having at least one LLIN compared to control village (OR =2. 2, P <0.001). More households reported owning only one LLIN than two or more, in contrast to the control household (OR = 2.1, P <0.001) and close to two-thirds of GG households bought their LLINs, whether subsidized or not, unlike control households where half obtained their LLINs free (OR = 0.63, P <0.001). The mean number of LLINs owned was 1.6 for GG and 1.8 for control village, with a mean difference of -0.26 (95% CI = - 0.34, -0.19; SE = 0.04). Fewer respondents from GG replied that they had perceivably sufficient LLINs for their family members compared to control villages (OR = 0.24, P <0.001); however, fewer of GG residents reported intention of having additional LLINs in the future compared to control villages (OR = 0.61) but the difference was not statistically significant (P = 0.22). The majority (95.7%) of GG households were expecting to receive LLINs for free, whereas three-quarters of the control households reported the hope of obtaining free LLINs sufficient for the needs of their family members in the future. Only a small proportion (4.2% of GG, 23.3% of control) had the intention to buy LLINs for their families. The average suggested cost of an LLIN was 17.0 Birr (≈ $0.9) with median 10.0 Birr (lower and upper quartiles 6.0 and 20.0, respectively).

The reported utilization rate of LLINs was higher among control households than GG households. Of those households with at least one LLIN, more respondents from GG reported that at least one of their family members did not use bed net compared to control population (OR = 2.8, P <0.001). Similarly, the control population was twice likely (OR = 0.48, P <0.001) to have slept under a bed net the previous night and more than five times likely (OR = 0.19, P < 0.001) to have slept under a bed net in the last month preceding the survey, compared to respondents from GG (Table 2).

**Table 2:** Comparison of ownership and utilization of long-lasting, insecticide-treated bed nets between Gilgel-Gibe and control villages, Southwest Ethiopia, November 2012

**Long-lasting, insecticide -treated bed nets (LLINs)**		**Gilgel-Gibe villages**		**Control villages**		**OR (95% CI)**
		**N**	**%**	**N**	**%**	
Ownership of LLINs (at least one)	Yes	787	66.3	555	46.8	2.2 (1.9-2.6)*
	No	400	33.7	631	53.2	
Number of LLINs owned	One	418	35.2	194	16.4	2.1 (1.9 -3.8)*
	≥ Two	369	31.1	361	30.4	
Source of LLINs	Free	307	39.0	279	50.3	0.63 (0.51 – 0.79)*
	Bought	480	61.0	276	49.7	
Used last night (those who had ≥1)	Yes	760	60.1	778	76.1	0.47 (0.39-0.57)*
	No	505	39.9	245	23.9	
Used last month (those who had ≥1)	Yes	759	60.0	910	89.0	0.19 (0.15-0.23)*
	No	506	40.0	113	11.0	
Any one from the family member who did not use LLINs	Yes	454	57.7	182	32.8	2.8 (2.2- 3.5)*
	No	333	42.3	373	67.2	
Sufficient LLINs for family members (those who have ≥1)	Yes	329	41.8	415	74.8	0.24 (0.19-0.31)*
	No	458	58.2	140	25.2	
Intention to have LLINs in future (those who do not have)	Yes	841	97.9	762	98.7	0.61 (0.28, 1.3)*
	No	18	2.1	10	1.3	
Mechanisms of having LLINs	Bought	35	4.2	178	23.3	1.6 (0.2-13)
	Free	805	95.7	577	75.6	11.2 (1.4 – 89.5)*

*Significant at 0.05.

Factors associated to number of LLINs owned were assessed using multinomial logistic regression. Variables such as gender, educational status and occupational status of household heads did not show a significant association with ownership of LLINs. On the other hand, variables such as age of household heads, household relative wealth index (RWI), distance to nearest health service and accessibility to transportation (all-weather roads) did show a significant association with ownership of LLINs. The chance of having a bed net is positively associated with age of household heads; particularly the probability of having two or more was positively related to age of household heads. Those households with heads aged 60 years or above were nearly twice as likely to have one bed net and more than three-fold likely to have two or more, compared to families with head of household less than 30 years old. Families with head of household who were married were twice more likely to have two or more LLINs than those families whose heads were single, widowed or separated. Household relative wealth index did not show chance difference of having one bed net among the households of different wealth index quantile categories. On the other hand, the probability of having two or more bed nets was found to be directly associated with household wealth indices. Those households in the fifth quantile category were 3.3 and 1.7 times more likely to have two or more LLINs compared to those households in the first and second quantile categories, respectively.

Households within 1 km of the nearest heath services were 12 times more likely to have one LLIN and 45 times more likely to have two or more LLINS compared to those households beyond 2 km from health services. Those households found from 1–2 km were seven times more likely to have one LLIN and nearly 12 times more likely to have two or more LLINS compared to those households beyond 2 km from health services. Similarly, those families who had perceivably accessibility to transportation were 1.5 times more likely to have one LLIN and twice as likely to have two or more LLINs compared to those who did not have access to transportation. Residents of GG were seven times and four times more likely to have one LLIN and two or more LLINs than control residents, respectively. Finally, household size was found to be a predictor of the number of LLINs owned. Households with three or less members were more than three times more likely to have one LLIN but 1.4 less likely to have two or more LLINs. Households having four to six family members were twice as likely to have one LLIN compared to those households with seven or more family members; however, there was no a significant difference between them having two or more LLINs (Table 3).

**Table 3:** Factors associated to number of owned long-lasting, insecticide-treated bed nets by household, southwest Ethiopia, November 2012

**Variables**	**Variables categories**	**Total**	**Owned ≥ 1 LLINs**		**Owned 1 LLIN**	**Owned ≥ 2 LLINs**
		**N** **o** **(%)**	**N (%)**	**AOR (95% CI)**	**AOR (95% CI)**	**AOR (95% CI)**
Age of household heads	<30 years	356 (15.0)	165 (46.3)	0.4 (0.3, 0.6)**	0.6 (0.4, 0.9)*	0.3 (0.2, 0.4)**
	30-39 years	710 (29.9)	401 (56.5)	0.7 (0.5, 0.9)**	0.8 (0.6, 1.2)	0.6 (0.4, 0.9)**
	40-49 years	548 (23.1)	306 (55.8)	0.7 (0.5, 0.9)*	0.7 (0.5, 1.0)	0.7 (0.5, 0.98)*
	50-59 years	358 (15.1)	218 (60.9)	0.8 (0.8,1.5)	0.9 (0.5, 1.3)	0.8 (0.6, 1.1)
	≥ 60 years	401 (16.9)	252 (62.8)	1	1	1
Marital status	Married	2,080 (87.7)	1,186 (57.0)	1.3 (0.9, 1.9)	1.0 (0.8,1.5)	1.7 (1.1, 2.5)*
	Not married	293 (12.3)	156 (53.2)	1	1	1
Household RWI	Poorest	475 (20.4)	241 (50.7)	0.5 (0.4,0.7)**	0.8 (0.5, 1.1)	0.3 (0.2, 0.3)**
	Poor	461 (19.8)	256 (55.5)	0.7 (0.5, 0.99)*	1.0 (0.7, 1.5)	0.6 (0.4,0.8)**
	Middle	466 (20.0)	275 (59.0)	0.9 (0.7, 1.2)	1.1 (0.8, 1.7)	0.8 (0.6,1.1)
	Less poor	455 (19.5)	273 (60.0)	0.9 (0.7, 1.2)	1.0 (0.7, 1.5)	0.8 (0.6, 1.1)
	Least poor	472 (20.3)	268 (56.8)	1	1	1
Distance to the nearest health service	<1 km	1,655 (69.7)	1,051 (63.5)	21.9 (14.9, 32.2 )**	12.4 (8.0,19.2)**	45.1 (24.8, 82.1)**
	1-2 km	490 (20.6)	244 (49.2)	8.5 (5.7, 12.7)**	7.0 (4.4, 11.0)**	11.8 (6.3, 21.9)**
	>2 km	228 (9.6)	47 (20.6)	1	1	1
Accessible to transport	Yes	918 (38.7)	621 (67.6)	2.1 (1.8, 2.5)**	1.5 (0.8, 1.3)	2.1 (1.6, 2.6)**
	No	1,455 (61.3)	721 (49.6)	1	1	1
Residence	GG area	1,187 (50.0)	787 (66.3)	1.8 (1.5, 2.2)**	7.0 (5.5, 9)**	4.3 (3.4, 5.5)**
	Control area	1,186 (50.0)	555 (46.8)	1	1	1
Household size	≤3 members	638 (26.9)	338 (53.0)	0.99 (0.7, 1.3)	3.4 (2.3, 5.0)**	0.3 (0.2, 0.5)**
	4-6 members	1,122 (47.3)	654 (58.3)	1.1 (0.9, 1.4)	2.1 (1.5, 3.0)**	0.9 (0.7, 11)
	≥7 members	613 (25.8)	350 (57.1)	1	1	1

*significant at 0.05 and **significant at 0.001.

Regarding utilization of LLINs, factors such as gender, educational and occupational status of household heads were not related to utilization of LLINs. Age, marital status of household heads, relative wealth index, distance to nearest health service, accessibility to transport, residence and household size were significantly associated with the utilization of LLITS. As age of household heads increased, utilization of LLINs showed steady decline. Households with heads less than 30 years and those with household heads of 30 to 49 years reported to have used LLINs; respectively, more than three times and about twice as likely compared to those households with household heads of 60 years or older. However, a significant difference was not seen between households with household heads age 50 years and above. Household heads who were married reported more than three times the use of LLINs compared to households whose heads were widowed, divorced or separated.

Household relative wealth index was found to be an influential predictor of use of LLINs. Wealthy households reported more use of LLINs. The wealthiest families were more than three times more likely to use LLINs than poor families. The least poor households were 2.5 and 1.7 times more likely to use LLINs compared to middle and less poor families, respectively. Households within 1 km radius of health services were 17.5 times, and those within 1–2 km from health services were 6.7 times more likely to use LLINs. Households in the control villages were twice as likely to use LLINs more than households around GGHD. One last influential predictor is household size, where those households with three or fewer members reported more use of LLINs than households with large families (Table 4).

**Table 4:** Factors associated with long-lasting, insecticide-treated bed net utilization, southwest Ethiopia, November 2012

**Variable**	**Categories**	**Used LLINs N (%)**	**AOR (95% CI)**
Age head of household	<30 yrs	148 (69.8)	3.5 (1.6, 7.6)**
	30-39 yrs	461 (69.5)	2.1 (1.3, 3.6)**
	40-49 yrs	403 (69.5)	1.8 (1.1, 3.0)*
	50-59 yrs	253 (64.9)	1.4 (0.8, 2.4)
	≥60 yrs	255 (63.0)	1
Gender of household head	Male	1,313 (68.5)	1.1 (0.9, 1.5)
	Female	207 (62.3)	
Marital status ofhousehold head	Married	1,384 (67.9)	3.6 (1.0, 12.4)*
	Not married	133 (64.3)	4.6 (0.6, 30.3)
Educational status of household head	None	1,126 (67.7)	1 (0.5, 2.0)
	Read and write	148 (72.9)	1.2 (0.6, 2.7)
	Elementary (1.4)	128 (60.7)	0.6 (0.3, 1.2)
	Elementary (5–8)	75 (64.7)	0.6 (0.3, 1.5)
	Grade ≥ 9	40 (76.9)	1
Occupational status of household head	Farmer	893 (49.0)	0.5 (0.2, 1)
	Housewife	319 (84.4)	0.6 (0.3, 1.2)
	Daily labourer	12 (46.2)	0.8 (0.3, 2.4)
	Others^1^	41 (67.2)	1
RWI of household	Very poor	182 (55.6)	0.3 (0.2, 0.6)**
	Poor	255 (63.3)	0.3 (0.2, 0.5)**
	Middle	310 (67.5)	0.4 (0.3, 0.7)**
	Less poor	345 (70.7)	0.6 (0.4, 0.9)
	Least poor	395 75.7	1
Distance to health services	<1 km	942 (50.9)	17.5**
	1-2 km	260 (69.1)	6.7**
	2 km	63 (100)	1
Access to transport	Yes	637 (57.2)	0.3 (0.21, 0.33)**
	No	880 (77.8)	1
Residence	Gilgel-Gibe villages	742 (60.3)	0.5 (0.3, 0.7)**
	Control villages	778 (76.3)	
Family size	1-3 members	288 (69.4)	1.7 (1.2, 2.3)**
	4-6 members	721 (66.3)	1.1 (0.9, 1.4)
	7 and above	511 (68.5)	1

^1^Others include: government employees (1.1%), merchants (0.8%), self-employed (0.7%) and students (0.3%).

*significant at 0.05 and **significant at 0.001.

## Discussion

This study has tried to assess possession and utilization of LLINs in households of rural communities. Attempts have been made to identify associated factors that influence the ownership and utilization level in the households, comparing the study sites on the basis of characteristics that can possibly make the difference. The coverage of LLINs was relatively higher among GG residents (66.3% *vs* 46.8%) than control villages. However, both were below the coverage recommended by WHO [17] for an acceptable level of protection. The ownership level was a similar average figure indicated in World Malaria Report of 2011 (median = 56%) [17] from household survey results.

The perceived utilization of LLINs among those households who had at least one LLIN was higher among the controls (76.1 *vs* 60.1%). The overall level of utilization of LLINs was similar to other local findings [12,32] which were below WHO recommendation of 80% utilization. In about 58% of GG and 33% of the control village households, at least one person did not sleep under a bed net and similar proportions of households reported respectively that the existing LLINs were not sufficient for their family members. Most of those households expected free distribution of LLINs and only a small proportion of them had the intention to buy LLINs for their families. The average suggested cost of LLINs was 17.0 Birr (≈ $0.9) with median 10.0 Birr which is similar to a previous finding where nearly half of study participants suggested 10 Birr or lower and the rest above 10.0 Birr for an ITN [33]. This is far below what others reported both from local [34,35] and some African countries [36-38]. The fact that control households were more ready to buy and reported more utilization than GG households implies that the control households were more aware of the benefits of LLINs.

Households with older heads possessed more LLINs, particularly when the number of LLINs owned was considered, but utilization was higher among younger heads. Household relative wealth index was positively associated both to possession and utilization of LLINs as possibly the wealthiest were able to access information to seek and receive or buy LLINs. This is a similar finding where the household wealth status was positively associated with ownership of LLINs [16]. Household wealth index was not found to be an important predictor of number of LLINs owned as the possession of one LLIN was similar for all and only the first and second quantiles were differently disadvantaged to have two or more LLINs. This finding is in agreement with other studies [39-42] where inequalities were observed in the number of ITNs owned and average number of ITNs possessed in which case the poorest were disadvantaged; but this is in contrast to another study [43] which failed to show a difference among socio-economic strata with respect to possession and likelihood of receiving free ITNs. Another study reported neither socio-economic nor educational level of mothers was associated with ITN utilization by children [44].

Similarly, those households who were nearer to health services and accessible to transportation were better by far than those distant and inaccessible households both in ownership and utilization of LLINs, especially when the number of owned LLINs was considered, nearness to health services is the most influential factor. The importance of distance to nearest health services or markets (possibly due to transportation accessibility), homestead wealth and residences had been identified by other studies [40,41,45] to account for the variation in utilization of LLINs. Unlike other findings [41,42], factors such as gender, educational, occupational, marital status of household heads and family size did not show significant difference both in possession and utilization of LLINs in this study. However, Nektiah-Amponsah [45] reported that the aforementioned variables significantly affect the adoption and utilization of ITNs among children under five years of age.

## Conclusion

Disparities have been seen among households both in ownership and utilization. The poorest, distant from health services, inaccessible to vehicle transportation households were found to be the most disadvantaged in possession of LLINs. Important factors that adversely influence utilization of LLINs were household RWI, distance to health services, inaccessibility to vehicle transportation, being in the control area, larger family size, household with heads that were not in marriage, and households with older heads.

Therefore the investigators suggest that attention needs to be given to the poor, distant and inaccessible households in the efforts of malaria intervention programmes, such as during free distribution of LLINs. Distribution of LLIN programmes should give due consideration to family size. Well-tailored information, education and communication (IEC) is needed to address the problem of non-users, especially households with older heads.

## Abbreviations

AOR: Adjusted odds ration; FMOH: Federal Ministry of Health; GG: Gilgel-gibe; GGHD: Gilge-gibe hydroelectric dam; IEC: Information, education and communication; ITNs: Insecticide-treated bed nets; LLINs: Long-lasting insecticide-treated bed nets; SPSS: Statistical package for social sciences; WHO: World Health Organization.

## Competing interests

The authors declare that they have no competing interests.

## Authors’ contributions

LDS designed the study, undertook the field study, analysed data and wrote the manuscript. WAD designed the study, revised the manuscript and facilitated administrative issues. AAA designed the study, revised the manuscript and facilitated administrative issues. All authors read and approved the final manuscript.
